# Intolerance of uncertainty and attitudes towards persons living with disabilities in medical students: Is there a correlation?

**DOI:** 10.3389/fpubh.2023.1149725

**Published:** 2023-03-24

**Authors:** Martinique Ogle, Dimitrios Papanagnou, Kestrel Reopelle, Frances Rusnack, Jordan Feingold-Link, Maria Poluch, Nethra Ankam

**Affiliations:** Sidney Kimmel Medical College (SKMC), Philadelphia, PA, United States

**Keywords:** uncertainty, disability, curriculum, medical education, student attitude surveys, medical student

## Abstract

**Introduction:**

Patients living with a disability experience an illness trajectory that may be uncertain. While navigating clinical uncertainty has been well-researched, health professionals’ intolerance of uncertainty for patients living with disabilities has yet to be explored. We examined the relationship between medical students’ intolerance of uncertainty with their attitudes towards people living with disabilities to better inform curricular efforts.

**Methods:**

We employed a survey-based design consisting of the Intolerance of Uncertainty Scale (IUS) and Disability Attitudes in Healthcare (DAHC) Scale to medical students upon completion of core clerkships (end of third-year of training). Data were de-identified. Mean DAHC and IUS scores were compared with published values *via t*-test. Linear regression was used to examine IUS/DAHC scores for anonymized students. Pearson correlation coefficient was calculated to assess correlation between IUS and DAHC scores.

**Results:**

Response rate was 97% (268/275 students). Mean IUS score did not differ from previously cited medical student scores, but mean DAHC score was significantly higher than previously cited scores. We observed a statistically-significant relationship between IUS and DAHC scores. Students with greater intolerance of uncertainty had lower scores for disability attitudes [*F*(1,243) = 8.05, value of *p* < 0.01], with an *R*^2^-value of 0.032, suggesting that 3% of DAHC score variance can be explained by IUS score changes.

**Conclusion:**

We identified a weak negative correlation between IUS and DAHC scores in medical students. Further research is needed to clarify findings and identify best practices that equip trainees with skills to care for patients with uncertain illness trajectories and patients living with disabilities.

## Introduction

Navigating uncertainty in clinical practice has been well-described across various clinical environments and specialties (1–3), but little research has specifically examined health professionals’ clinical uncertainty in managing the care trajectories of patients living with disabilities. A gap remains in exploring the relationship between providers’ tolerance of uncertainty and their attitudes towards patients living with one or more cognitive and/or developmental disabilities.

Clinicians with a higher intolerance of uncertainty are more likely to be burned out, less likely to be engaged at work (1), and physicians who face anxiety due to uncertainty are less likely to engage in shared decision-making when compared to colleagues with less uncertainty-induced anxiety (3). Post-graduate trainees have also shared a desire for additional education that prepares them to managing their uncertainty, with a preference for this training to begin as early as in medical school (4). Fortunately, there are a range of medical education interventions that have reported a positive impact on learners’ uncertainty tolerance, as well as their emotional responses to the uncertainty itself (5).

To further explore this gap, we built upon our previous work evaluating medical students’ intolerance of uncertainty. We specifically explored the relationship between their intolerance of uncertainty and their attitudes towards persons living with disabilities. Over recent years, to address the discrimination and health disparities experienced by individuals living with disabilities, medical schools have integrated curricula that equip students with the skills to care for patients with disabilities and to identify ableism (6). Implicit bias against people living with one or more disabilities remains strongest in the United States (7, 8). Research examining this association would help inform educational interventions that better prepare trainees in the health professions to navigate uncertainty in clinical practice, with a specific focus on managing the uncertain care trajectories of patients living with disabilities. Moreover, this would serve as an ideal opportunity to further shine light on implicit biases against persons living with disabilities.

## Methods

### Study design

The study was conducted with third-year medical students at the end of core clinical clerkships who were about to begin senior sub-internships. A survey-based design was employed consisting of two validated psychometric instruments, with additional items to collect student demographics. The instruments were assigned as pre-session reflection exercises for required coursework on navigating uncertainty in clinical practice. The study was reviewed and approved by the Institutional Review Board at our university.

### Participants

Participants were third-year medical students at the end of core clinical clerkships at a large, urban medical school in Philadelphia, Pennsylvania.

### Instruments

We chose to measure students’ attitudes towards persons living with disabilities using the Disability Attitudes in Healthcare (DAHC) Scale, a 17-item Likert scale (9). The scores of this scale are composite and range from 17 to 85, with a higher score implying a more positive attitude towards patients with disabilities (9). This scale has been previously used by medical students at large urban medical schools in the United States (9, 10). The Cronbach alpha test for the DAHC scale is 0.82, indicating good internal consistency.

We also measured students’ intolerance of uncertainty using the short version of the Intolerance of Uncertainty Scale (IUS), a 12-item psychometrically-validated instrument for measuring intolerance of uncertainty (11). The scores of this scale are also composite and range from 12 to 60, with a higher score indicating a greater intolerance of uncertainty. This scale has previously been used by medical students at our medical school (12, 13). The Cronbach alpha test for the IUS scale is 0.87, indicating good internal consistency.

### Survey administration

A survey link was emailed to 275 third-year medical students to complete as pre-session work 1 week prior to coursework on navigating uncertainty in clinical practice. The goal of this pre-session work was to allow students to reflect on their attitudes prior to attending the workshop. Completion of the survey was completely voluntary. Qualtrics software (Qualtrics, Provo, UT) was used to administer the online survey. Responses were automatically anonymized during completion of the survey by the Qualtrics software. No identifying information was collected.

### Data analysis

Survey data were exported into Microsoft Excel 2016 (Microsoft Corp, Redmond, WA) for analysis. A linear regression was run between the IUS scores and corresponding DAHC scores generated by each student. An F-test was completed to assess the best-fit line of the linear regression. The Pearson correlation coefficient was also calculated to assess correlation between the IUS and the DAHC scores. Data were de-identified, and mean DAHC and IUS scores were compared with published values *via t*-test. All methods used in the data analyses used a critical level (alpha) of 0.05.

## Results

Two hundred sixty-eight medical students completed the survey (response rate 97.45%, 268/275), of which 246 students completed both the IUS and DAHC scales. The mean score for IUS did not differ from the previously reported mean IUS scores for medical students (29.76, SD 8.24 vs. 28.73, SD 8.65, respectively; *p* = 0.1717) (12). Mean scores for DAHC did differ from previously reported mean scores for medical students (70.5, SD 7.5 vs. 64.8, SD 8.1, respectively; *p* < 0.0001) (9) and did differ from our internal data, previously collected at our medical school in 2013 (70.5, SD 7.5 vs. 63.10, SD 6.6, respectively, *p* < 0.0001).

**Figure 1:**
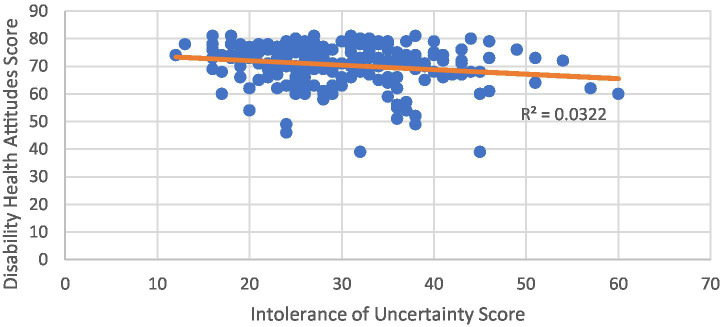
Linear regression between intolerance of uncertainty scale (IUS) and disability attitudes in healthcare (DAHC) scores.

A linear regression was also calculated to evaluate the relationship between IUS score and DAHC score. There was a statistically significant relationship between IUS and DAHC scores [*F*(1,243) = 8.05, value of *p* < 0.01] with an *R*^2^ value of 0.032. The Pearson correlation test for the DAHC and IUS relationship was −0.18 (value of *p* < 0.005), indicating a statistically significant negative correlation (Figure 1).

## Discussion

Our results suggest a negative correlation between IUS and DAHC scores in medical students at the completion of core clinical clerkships. The small *R*^2^ value, however, suggests that only approximately 3% of the variance of the DAHC scores can be explained by changes in IUS score. While it is encouraging to find this correlative relationship between these two scales, we are aware of the limitations we can draw from this data.

While student DAHC scores were higher than previously cited scores, this observation may possibly be explained by social desirability bias, which has been previously observed in subjects who positively overestimate their own attitudes towards patients with disabilities and, thus, have artificially inflated scores (14). Changes in the public education system have been suggested to explain this bias. A Polish study observed that people ages 19–34 had more positive perceptions of people with disabilities than middle-aged individuals, and suggested that a fully-integrative and inclusive elementary and secondary education may have influenced this observation in younger individuals (15). This may have influenced our results in US medical students. The US Department of Education reports that only 31.7% of students aged 6–21 with disabilities spent at least 80% of their time in general classes in a regular school in 1989, but 64.0% of this population was in general classes in a regular school for at least 80% of their day by 2018 (16). Current medical students who completed K-12 American public education were likely exposed to peers with disabilities, and may have impacted their perception of persons living with disabilities. This relationship, of course, requires further investigation.

A major limitation of our findings stems from surveying medical students from one medical school at a single point in time, which may affect generalizability. However, to our knowledge this is the first study to examine the intersection of uncertainty tolerance and attitudes towards persons living with disabilities. We also recognize the limitations in examining students’ uncertainty tolerance, as “competence in managing complex cases does not necessarily arise through the development of uncertainty tolerance” (17). Rather tolerance for uncertainty in clinical practice has the potential to develop as clinicians develop their skills to manage complex patients.

While our study did show a significant relationship between medical students’ intolerance of uncertainty and attitudes towards patients with disabilities, intolerance of uncertainty seems to have limited influence on attitudes towards persons living with disabilities. Further research is needed to clarify these findings and identify best practices to equip medical students with the skills to care for patients with uncertain illness trajectories. Such work is needed to develop best practices in a curriculum that prepares trainees to care for patients living with disabilities.

## Data availability statement

The raw data supporting the conclusions of this article will be made available by the authors, without undue reservation.

## Ethics statement

The studies involving human participants were reviewed and approved by Institutional Review Board of Thomas Jefferson University. Written informed consent for participation was not required for this study in accordance with the national legislation and the institutional requirements.

## Author contributions

DP, MO, KR, NA, and FR: writing of manuscript. JF-L, MO, and MP: data analysis. NA: project oversight and content expertise. DP, FR, and KR: editing. All authors contributed to the article and approved the submitted version.

## Conflict of interest

The authors declare that the research was conducted in the absence of any commercial or financial relationships that could be construed as a potential conflict of interest.

## Publisher’s note

All claims expressed in this article are solely those of the authors and do not necessarily represent those of their affiliated organizations, or those of the publisher, the editors and the reviewers. Any product that may be evaluated in this article, or claim that may be made by its manufacturer, is not guaranteed or endorsed by the publisher.
